# Q&A: What is human language, when did it evolve and why should we care?

**DOI:** 10.1186/s12915-017-0405-3

**Published:** 2017-07-24

**Authors:** Mark Pagel

**Affiliations:** School of Biological Sciences, University of Reading, Reading, RG6 6UR UK

## Abstract

Human language is unique among all forms of animal communication. It is unlikely that any other species, including our close genetic cousins the Neanderthals, ever had language, and so-called sign ‘language’ in Great Apes is nothing like human language. Language evolution shares many features with biological evolution, and this has made it useful for tracing recent human history and for studying how culture evolves among groups of people with related languages. A case can be made that language has played a more important role in our species’ recent (circa last 200,000 years) evolution than have our genes.

## What is special about human language?

Human language is distinct from all other known animal forms of communication in being *compositional*. Human language allows speakers to express thoughts in sentences comprising subjects, verbs and objects—such as ‘I kicked the ball’—and recognizing past, present and future tenses. Compositionality gives human language an endless capacity for generating new sentences as speakers combine and recombine sets of words into their subject, verb and object roles. For instance, with just 25 different words for each role, it is already possible to generate over 15,000 distinct sentences. Human language is also *referential*, meaning speakers use it to exchange specific information with each other about people or objects and their locations or actions.

## What is animal ‘language’ like?

Animal ‘language’ is nothing like human language. Among primates, vervet monkeys (*Chlorocebus pygerythrus*) produce three distinct alarm calls in response to the presence of snakes, leopards and eagles [1]. A number of parrot species can mimic human sounds, and some Great Apes have been taught to make sign language gestures with their hands. Some dolphin species seem to have a variety of repetitive sound motifs (clicks) associated with hunting or social grouping. These forms of animal communication are symbolic in the sense of using a sound to stand in for an object or action, but there is no evidence for compositionality, or that they are truly generative and creative forms of communication in which speakers and listeners exchange information [2].

Instead non-human animal communication is principally limited to repetitive instrumental acts directed towards a specific end, lacking any formal grammatical structure, and often explainable in terms of hard-wired evolved behaviours or simple associative learning [2]. Most ape sign language, for example, is concerned with requests for food. The trained chimpanzee Nim Chimpsky’s longest recorded ‘utterance’, when translated from sign language, was ‘give orange me give eat orange me eat orange give me eat orange give me you’ [3]. Alarm calls such as observed in the vervet monkeys often evolve by kin-selection to protect one’s relatives, or even selfishly to distract predators away from the caller. Hunting and social group communications can be explained as learned coordinating signals without ‘speakers’ knowing why they are acting as they are.

## When did human language evolve?

No one knows for sure when language evolved, but fossil and genetic data suggest that humanity can probably trace its ancestry back to populations of anatomically modern *Homo sapiens* (people who would have looked like you and me) who lived around 150,000 to 200,000 years ago in eastern or perhaps southern Africa [4–6]. Because all human groups have language, language itself, or at least the capacity for it, is probably at least 150,000 to 200,000 years old. This conclusion is backed up by evidence of abstract and symbolic behaviour in these early modern humans, taking the form of engravings on red-ochre [7, 8].

The archaeological record reveals that about 40,000 years ago there was a flowering of art and other cultural artefacts at modern human sites, leading some archaeologists to suggest that a late genetic change in our lineage gave rise to language at this later time [9]. But this evidence derives mainly from European sites and so struggles to explain how the newly evolved language capacity found its way into the rest of humanity who had dispersed from Africa to other parts of the globe by around 70,000 years ago.

## Could language be older than our species?

Ancient DNA reveals us to be over 99% identical in the sequences of our protein coding genes to our sister species the Neanderthals (*Homo neanderthalensis*) [10]. The Neanderthals had large brains and were able to inhabit much of Eurasia from around 350,000 years ago. If the Neanderthals had language, that would place its origin at least as far back as the time of our common ancestor with them, currently thought to be around 550,000 to 750,000 years ago [10, 11].

However, even as recently as 40,000 years ago in Europe, the Neanderthals show almost no evidence of the symbolic thinking—no art or sculpture for example—that we often associate with language, and little evidence of the cultural attainments of *Homo sapiens* of the same era. By 40,000 years ago, *Homo sapiens* had plentiful art, musical instruments and specialized tools such as sewing needles. Neanderthals probably didn’t even have sewn clothing, instead they would have merely draped themselves with skins [12]. And, despite evidence that around 1–5% of the human genome might be derived from human–Neanderthal matings [13], the Neanderthals went extinct as a species while we flourished.

## Can genetic evidence help to decide when language evolved?

Yes. Modern humans and Neanderthals share a derived version of a transcription factor gene known as *FOXP2* that differs from the chimpanzee version by two amino acid replacements [14]. *FOXP2* influences the fine-motor control of facial muscles required for the production of speech. Indeed, inserting this derived form into mice causes them to squeak differently [15]! However, in spite of having identical primary sequences to Neanderthals, modern humans have acquired changes to the regulation of their *FOXP2* genes that seem likely to cause their *FOXP2* to be expressed differently to that of the Neanderthals [16], and these expression differences are pronounced in brain neurons. Combining these genetic hints with the differences in symbolic and cultural behaviour that are evident from the fossil record suggests language arose in our lineage sometime after our split from our common ancestor with Neanderthals, and probably by no later than 150,000 to 200,000 years ago.

## Was there a single origin of language?

This question has parallels in biological evolution. Did life evolve once or many times? The presence of the same RNA and DNA in all organisms and homologies in the machinery of DNA transcription and translation suggest that at least all current life on Earth has a common origin. It is possible that life evolved more than once but all descendants of these other origins went extinct and left no fossil or other traces.

With language the inference is harder to make because features such as vocabulary and grammar change too rapidly to be able to link all of the world’s languages to a common original mother tongue. On the other hand, all human languages rely on combining sounds or ‘phones’ to make words, many of those sounds are common across languages, different languages seem to structure the world semantically in similar ways [17], all human languages recognize the past, present and future and all human languages structure words into sentences [18]. All humans are also capable of learning and speaking each other’s languages (some phones are unique to some language families—such as the famous ‘click’ sound of some San languages of Southern Africa—but these are probably within the capability of all human speakers if they are exposed to learning that sound at the right time of life).

These considerations suggest that the anatomical, neurological and physiological underpinnings of language are shared among all of humanity. If the capacity for language did evolve more than once, all traces of it seem to have been lost. This conclusion is buttressed by the *FOXP2* evidence (all humans share the same derived gene) and by the fact that genetic data point to all modern humans descending from a common ancestor [19].

## Is language evolution like biological evolution?

Darwin observed that “The formation of different languages and of distinct species, and the proofs that both have been developed through a gradual process, are curiously the same” (page 59 in [20]). He also asserted that “The survival and preservation of certain favoured words in the struggle for existence is natural selection.” (pages 59–60 in [20]).

Darwin was right on both counts. Linguists have known from at least the late 18th century [21]—about 100 years before Darwin—that languages predominantly evolve by a process of descent with modification from earlier ancestral languages, just as biological species descend from earlier ancestral forms. An example is differences observed between the ancient Greek vocabulary in Homer’s Iliad from around 750 BCE and modern Greek vocabulary (Table 1) [22]: some words have merely changed their pronunciation while others have been replaced by new unrelated words.

**Table 1:** Linguistic descent with modification spanning nearly three millennia^a^

Word	Homeric Greek	Modern Greek
One	eis (είς)	ena (ένα)
Day	emar (ήμαρ)	emera (ημέρα)
Two	dyo (thuo) (δύο, δύω)	dyo (δυο)
Father	pater (πατήρ)	pateras (πατέρας)
To eat	**etho** (έδω)	faei (φάει)
Bird	**ornis** (όρνις)	pouli (πουλί)

^a^Homeric Greek of the Iliad dates from ~750 BCE. Words in bold have been replaced in Modern Greek

Regarding Darwin’s assertions that certain words are favoured in the ‘struggle for existence’, it is useful to remember that there is seldom any connection between a sound (a word) and its meaning. This means that selection is reasonably free to choose among words and so features of the words we actually use might reveal its actions. The simplest example is that words that are used more often—such as *I*, *he*, *she*, *it*, *the*, *you*—tend to be shorter, and consequently easier to pronounce, than less frequently used words, such as *obstreperous* or *catafalque* [23]. This is an example of a form of natural selection except here instead of biological individuals competing in the physical environment to survive and reproduce, words compete for space in the environment of the human mind. Our minds give preference to shorter versions of the frequently used words, presumably to reduce effort [23]. This pressure is relaxed among the less frequently used words, allowing them to be longer. It might also be the case that once the frequently used words have occupied the space of possible short words, there are fewer opportunities for the less frequently used words [24].

## Is it possible to reconstruct the history of a group of languages like we do with species?

**Fig. 1.**
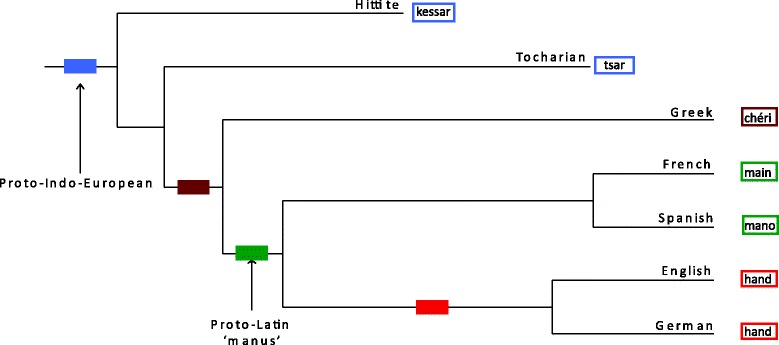
Phylogenetic tree of a small subset of the approximately 400 or so Indo-European languages. Words that the languages use for the meaning ‘hand’ are colour-coded to identify cognate classes. *Rectangles* along the branches identify regions of the tree where new cognate classes might have arisen. Here the French and Spanish languages share cognate forms for ‘hand’ derived from an earlier Latin form ‘manus’. French and Spanish are part of the familiar grouping of Romance languages. By comparison, the word ‘hand’ is cognate between English and German and this cognate class identifies part of the Germanic grouping of languages. The words for ‘hand’ in Greek and in the extinct Anatolian languages Hittite and Tocharian form two additional cognate sets. Combining many different cognate sets from many different vocabulary items allows investigators to draw detailed phylogenetic trees of entire language families (see text)

Yes. Using common lists of words that are found in all or nearly all languages, linguists can identify shared sets of *cognate* words—words that descend from common ancestral words— just as it is possible to identify *homologous* genes that share a common ancestral gene. For instance the Spanish *mano* (‘hand’) and the French *main* descend from the earlier Latin *manus*, while the English and German words *hand* do not. A cognate set identifies groups of related languages. In the example here *mano* and *main* identify the so-called Romance languages (Spanish, French, Italian, Portuguese) and *hand* and *hand* identify the Germanic languages (Fig. 1). By combining the information in many different cognate sets with appropriate statistical models [25, 26], it is possible to infer detailed family histories or phylogenetic trees of language families, such as has been done for the Indo-European languages (Fig. 1). These phylogenies are directly analogous to phylogenies of biological species.

## What other evolutionary features do genes and language share?

Linguistic and biological evolution share features beyond descent with modification and selection, including mechanisms of mutation and replication, speciation, drift and horizontal transfer (Table 2). At a deeper level, both genes and languages can be represented as digital systems of inheritance, built on the transmission of discrete chunks of information—genes in the case of biological organisms, and words in the case of language. Genes in turn comprise combinations of the four bases or nucleotides (A, C, G, T) while words can be modelled as comprising combinations of discrete sounds or phones (in fact, phones or sounds vary in a continuous space but languages are commonly represented as expressing a particular set of discrete phonemes).

**Table 2:** Some parallels between biological and linguistic evolution

Biological evolution	Language evolution
Discrete heritable units (for example, nucleotides, amino acids and genes)	Discrete heritable units (for example, words, phonemes and syntax)
DNA copying	Teaching, learning and imitation
Mutation (for example, many mechanisms yielding genetic alterations)	Innovation (for example, formant variation, mistakes, sound changes, and introduced sounds and words)
Homology	Cognates
Natural selection	Social selection and trends
Drift	Drift
Speciation	Language or cultural splitting
Concerted evolution	Regular sound change
Horizontal gene transfer	Borrowing
Hybridization (for example, horse with zebra and wheat with strawberry)	Language Creoles (for example, Surinamese)
Geographic clines	Dialects and dialect chains
Fossils	Ancient texts
Extinction	Language death

These similarities mean that we can—and should—think of language as a system for the transmission of information that is tantamount to ‘aural DNA’. Even the peculiar phenomenon of concerted evolution in genetics—where a nucleotide replacement at a specific site in one gene is quickly followed by the same nucleotide replacement at the same site in other, typically related, genes—is also observed in language. Known as *regular sound change*, a specific phone or sound changes over a relatively short period of time to the same other phone in many words in the lexicon [27, 28]. A well-known example is the *p → f* sound change in the Germanic languages where an older Indo-European *p* sound was replaced by an *f* sound, such as in *pater → father*; or *pes, pedis → foot*.

## Can changes to language be used to trace human history?

There are currently about 7000 languages spoken around the world, meaning that, oddly, most of us cannot communicate with most other members of our species! Even this number is probably down from the peak of human linguistic diversity that was likely to have occurred around 10,000 years ago, just prior to the invention of agriculture [29]. Before that time, all human groups had been hunter-gatherers, living in small mobile tribal societies. Farming societies were demographically more prosperous and group sizes were larger than among hunter-gatherers, so the expansion of agriculturalists likely replaced many smaller linguistic groups. Today, there are few hunter-gatherer societies left so our linguistic diversity reflects our relatively recent agricultural past.

Phylogenies of languages can be used in combination with geographical information or information on cultural practices to investigate questions of human history, such as the spread of agriculture. Phylogenies of language families have been used to study the timing, causes and geographic spread of groups of farmers/fishing populations, including the Indo-Europeans [30–33]; the pace of occupation of the Pacific by the Austronesian people [34]; and the migration routes of the Bantu-speaking people through Africa [35, 36].

Linguistic phylogenies are also used to investigate questions of human cultural evolution, including the evolution and spread of dairying [37–39], relationships between religious and political practices [40], changing political structures [41] and the age of fairy tales [42], and have even supplied a date for Homer’s Iliad [22].

## What role has language played in our species’ success?

Language has played a prominent and possibly pre-eminent role in our species’ history. Consider that where all other species tend to be found in the environments their genes adapt them to, humans can adapt at the cultural level, acquiring the knowledge and producing the tools, shelters, clothing and other artefacts necessary for survival in diverse habitats [12, 43]. Thus, chimpanzees are found in the dense forests of Africa but not out on the savannah or in deserts or cold regions; camels are found in dry regions but not in forests or mountaintops, and so on for other species. Humans, on the other hand, despite being a species that probably evolved on the African savannahs, have been able to occupy nearly every habitat on Earth. Our behaviour is like that of a collection of biological species [43]. Why this striking difference?

It is probably down to language. Possessing language, humans have had a high-fidelity code for transmitting detailed information down the generations. Many, if not most, of the things we make use of in our everyday lives rely on specialized knowledge or skills to produce. The information behind these was historically coded in verbal instructions, and with the advent of writing it could be stored and become increasingly complex.

Possessing language, then, is behind humans’ ability to produce sophisticated cultural adaptations that have accumulated one on top of the other throughout our history as a species. Today as a result of this capability we live in a world full of technologies that few of us even understand. Because culture, riding on the back of language, can evolve more rapidly than genes, the relative genetic homogeneity of humanity in contrast to our cultural diversity shows that our ‘aural DNA’ has probably been more important in our short history than genes.
